# Mapping of dwarfing QTL of Ari1327, a semi-dwarf mutant of upland cotton

**DOI:** 10.1186/s12870-021-03359-x

**Published:** 2022-01-03

**Authors:** Chenhui Ma, Abdul Rehman, Hong Ge Li, Zi Bo Zhao, Gaofei Sun, Xiong Ming Du

**Affiliations:** 1 State Key Laboratory of cotton Biology, Institute of Cotton Research of the Chinese Academy of Agricultural Sciences, Anyang, 455000 China; 2 Zhengzhou Research Base, State Key Laboratory of Cotton Biology, Zhengzhou University, Zhengzhou, 450000 China; 3 Department of Plant Breeding and Genetics, Bahauddin Zakariya University, Multan, 66000 Pakistan; 4 State Key Laboratory of Cotton Biology, Research Base, Anyang Institute of Technology, Anyang, China

**Keywords:** Cotton, Semi-dwarf, QTL, Segregation distortion

## Abstract

**Background:**

Upland Cotton (*Gossypium hirsutum* L.) has few cotton varieties suitable for mechanical harvesting. The plant height of the cultivar is one of the key features that need to modify. Hence, this study was planned to locate the QTL for plant height in a ^60^Co γ treated upland cotton semi-dwarf mutant Ari1327.

**Results:**

Interestingly, bulk segregant analysis (BSA) and genotyping by sequencing (GBS) methods exhibited that candidate QTL was co-located in the region of 5.80–9.66 Mb at D01 chromosome in two F_2_ populations. Using three InDel markers to genotype a population of 1241 individuals confirmed that the offspring’s phenotype is consistent with the genotype. Comparative analysis of RNA-seq between the mutant and wild variety exhibited that *Gh_D01G0592* was identified as the source of dwarfness from 200 genes. In addition, it was also revealed that the appropriate use of partial separation markers in QTL mapping can escalate linkage information.

**Conclusions:**

Overwhelmingly, the results will provide the basis to reveal the function of candidate genes and the utilization of excellent dwarf genetic resources in the future.

**Supplementary Information:**

The online version contains supplementary material available at 10.1186/s12870-021-03359-x.

## Introduction

Cotton is an important cash crop worldwide, accounting for about 35% of the world’s fiber production [1]. Mechanical picking of cotton is a new trend of modern agriculture, but plant height (PH) is one of the most important factors which impede mechanical harvesting [2]. Nowadays, cotton plant height is controlled chemically, which adds additional cost and also leads to chemical contamination [3, 4]. Alternatively, the most reliable and feasible method is the use of natural semi-dwarf plants. The semi-dwarf traits can be used for mechanized management and increase lodging resistance and photosynthesis [5]. In contrast, the semi-dwarf natural resources of cotton are still limited due to fewer reports on the discovery of dwarf mutants and QTL (Quantitative Trait Loci) locations in related mutants.

Quisenberry et al. found a Lubbock dwarf mutant with poor fiber quality [6]. Abzalo et al. found a homozygous dominant lethal dwarf mutant line L-691, in cotton [7]. Moreover, some researchers also revealed that the phenotype of cotton mutants is closely linked with plant height [8, 9]. Others dwarf mutants showed extreme phenotypes without application potential. An ultra-dwarf mutant of cotton controlled by a du gene, and its dwarfing gene was located on chromosome A06 [10]. *GhLi-1* was a classical dwarf mutant of upland cotton, with short fibers. An SNP variant G65V affected its typical polymerization and biological processes based on the actin cytoskeleton (such as intracellular transport), which ultimately restrict cell elongation [11]. In cotton, *AS98* is a super-dwarf mutant with a plant height of 25.6 cm, and the candidate QTL was located between markers GH537 and E4M13 [12]. Recently, dwarf QTL was located in the F_2_ population of AS98 through BSA and revealed that dwarfness was caused by the copy number variation of *GhDREB1B* [13].

Plant height was provoked by multiple developmental factors, i.e., the number of phytomeres, stem length, and plant hormones [14]. Dwarf mutant *sd1* of rice was regulated by GA (Gibberellic Acid), which exhibited that plant hormone like GA plays a vital role in the green revolution and plant height control [15]. Negatively regulated GA-*DELLA* protein can interact with many transcription factors and ultimately affect cell elongation. Rice *SLR1* (DELLA) protein can directly interact with transcription factors *NAC29* and *NAC31*, which mediate *MYB61* and *cellulose synthase* genes [16]. In Arabidopsis stem tips, the *DELLA* protein inhibited several TCP transcription factors involved in the cell cycle [17]. Proteomic analysis of upland cotton revealed that its dwarfness might be regulated by GA pathways [18]. BR (Brassinosteroid) is another plant hormone that controls plant height by regulating cell elongation. Moreover, it was also noticed that disturbance in BR biosynthesis or signaling pathway reduce plant height significantly in rice and Arabidopsis mutants [19, 20]. IAA (Indole Acetic Acid) can control young leaves and roots apical meristem, promote root elongation, stem growth and flower differentiation. The IAA efflux transporter (PIN) family play a key role in plant height. Overexpression of both *ZmPIN1a* and *OsPIN2* can reduce plant height [21, 22]. Thiamin (vitamin B1) is involved in chloroplast photosynthesis and mitochondrial tricarboxylic acid cycle [23–25]. Research on various crop plants has shown that changes in thiamine levels affect plant height, yellowing of leaves, delay growth and development, delay flowering, and ultimately reduce yield [26–28].

With the development of sequencing technology, multiple high-throughput sequencing methods are widely used in QTL identification. Sun et al. successfully mapped 12 salt-tolerance-related genes using 196 upland cotton species from GWAS (Genome-Wide Association Studies) results and RNA-seq data [29]. 12 QTL related to lint percentage were located in 355 cotton genotypes by SLAF-GWAS in four environments and exhibited *Gh_A02G1268* responsible for lint percentage [30]. Map-based cloning is a research method to study the QTL of separated populations, but phenotypic values of the permanently separate population are essential to locate accurately in multiple environments [1, 31].

This study mapped semi-dwarf QTL of mutant cotton genotype Ari1327 precisely by GBS, BSA and RNA-seq database to locate the candidate gene area on the D01 chromosome. This work will provide a foundation to verify the dwarfing function of candidate genes and ultimately realize the use of excellent dwarfing resources.

## Results

### Phenotypic analysis

**Fig. 1:**
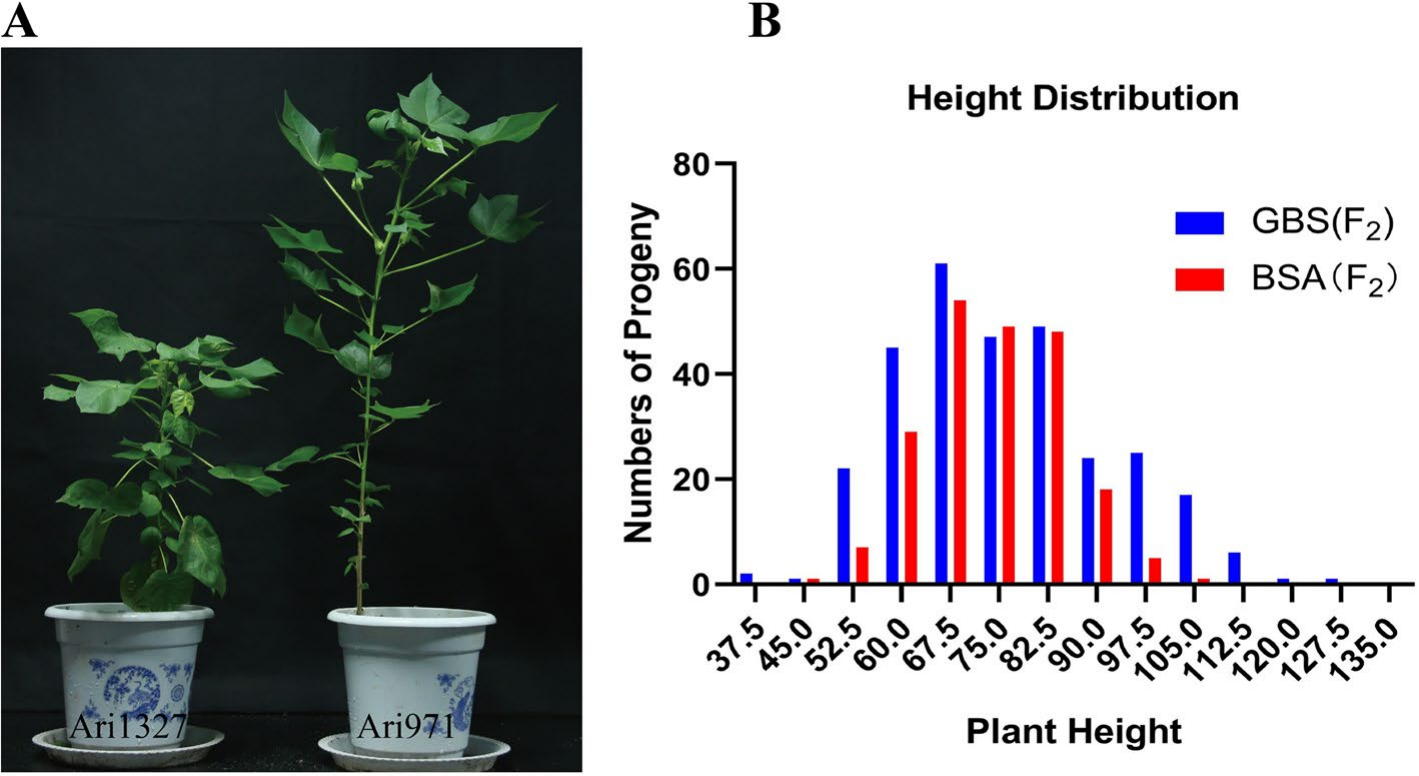
Phenotypic Analysis of Ari971, Ari1327 and F_2_ population. (A) The Plant height of dwarf mutant and wild type, left and right one are Ari137 and Ari971 respectively. (B) AA F_2_ (BSA) and BA F_2_ (GBS) group plant height distribution analysis

Ari1327 was a semi-dwarf mutant as compared to wild-type Ari971. Significant differences were observed between 16 agronomic and fiber traits for mutant and wild types in filed conditions. The results exhibited that mutant and wild type had plant height of 48.60 cm and 76.15 cm, respectively (Table 1 and Fig. 1A). The plant height of Ari1327 was only 68% of Ari971. Ari971 has 13 sympodial branches, 2.5 more than Ari1327. Among 11 fiber quality traits, only the average length of upper fibers (FL) revealed significant difference, while in mutant 7% reduction was also observed (*P* < 0.01). Among the five morphological data related to cotton growth, only the number of sympodial branches and plant height showed significant differences (P < 0.01).

**Table 1:** Statistical analysis of significance of morphological traits of cotton

Traits	Ari1327	Ari971	Significance
Average length of upper fibers (mm)	28.10 ± 0.95	30.07 ± 1.86	**
Boll Number	11.00 ± 2.75	11.45 ± 4.37	
Fiber Elongation	9.37 ± 1.59	9.46 ± 1.55	
Fiber Maturity	0.86 ± 0.02	0.84 ± 0.03	
Fiber Strength(cN/tex)	28.49 ± 2.68	30.85 ± 2.13	*
Fiber Uniformity	85.02 ± 1.28	85.99 ± 1.29	*
Fruit Branch Length	29.77 ± 10.97	33.80 ± 12.98	
Fruit branch number	10.50 ± 2.04	13.00 ± 1.45	**
Leaf area	145.10 ± 54.43	145.71 ± 24.96	
Leaf number	5.63 ± 2.58	5.79 ± 3.06	
Lint Percentage	36% ± 0%	40% ± 1%	*
Lint weight (30 Bolls)	52.75 ± 2.47	70.05 ± 1.91	*
Micronaire	4.97 ± 0.51	4.36 ± 0.84	*
Plant height	48.60 ± 11.54	76.15 ± 10.12	**
Seed Cotton weight (30 Bolls)	148.60 ± 8.34	176.55 ± 10.68	
Short Fiber (%)	5.92 ± 1.6	5.31 ± 0.86	

* mean significance, ** mean the differences are highly significant at the 0.05 and 0.01 level respectively

FL (average length of upper fibers), FU (fiber uniformity), FS (fiber strength), MC (micronaire), FE (fiber elongation) and PH (plant height) in the BA F_2_ population also revealed that fiber quality traits and plant height lack significant correlation (Additional file 1). Results also depicted that the correlation coefficient between PH and the other five fiber quality traits was ±0.15, with no significant correlation. While MC and FL, FU, FS showed a negative correlation. Moreover, the correlation between FL, FU, FS, and FE was higher than 0.45. Hence, it concluded that PH does not correlate with fiber quality traits hence, inherited independently.

Ari3697 (Group AA) and BL-Y10 (BA group) were used as two female parents to construct two F_2_ populations to identify the dwarfing QTL in Ari1327. Their PH values were 105.85 ± 7.81 cm and 93.70 ± 6.48 cm, respectively. The PH of male parent Ari1327 was 46.43 ± 5.57 and 67.36 ± 6.04 in the two populations. The number of individuals in the group AA and group BA were 214 and 303, respectively. The skewness of plant height of two populations was greater than 0, biased towards semi-dwarf parents. When the kurtosis is equal to 0, it predicts normal distribution characteristics. After the Shapiro-Wilk normality test of R software, the W values and *p*-values of the BA and AA groups were 0.99419, 0.97534 and 0.5844, 4.779e-05, respectively. It shows that the BA group confirmed the normal distribution (Fig. 1B, Additional file 9 and Additional file 10).

### BSA QTL locate in AA F_2_ population

**Fig. 2:**
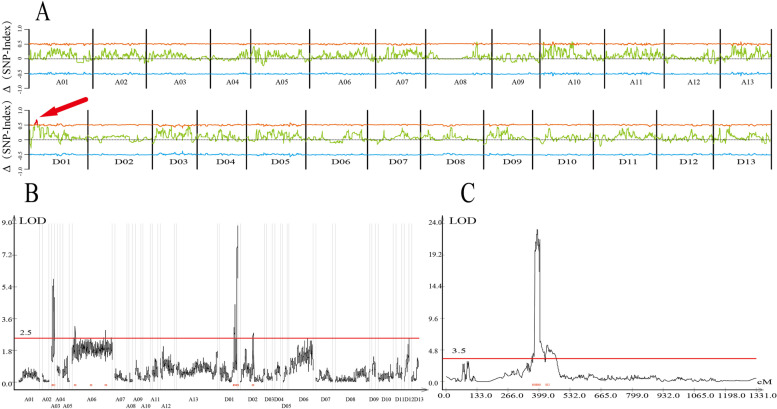
QTL-mapping in F_2_ populations. (A) BSA QTL-located for AA F_2_ group. The x-axis and y-axis represent the chromosome position and Δ (SNP-index) value, respectively. The line of red and blue indicate the 0.01 significance level. Only when the Δ (SNP-index) over this e-value, that it as the candidate QTL. (B) Map-based cloning for BA F_2_ group. The x-axis and y-axis represent the chromosome genetic position and LOD value, respectively. The line of red is LOD 2.5. (C) Map-based cloning for BA F_2_ group in new D01 linkage map alone. The x-axis and y-axis represent the D01 chromosome genetic position and LOD value, respectively. The line of red is LOD 3.5

BSA method was used to identify QTL of group AA with an e-value greater than 99%. A total of 10 QTLs were detected on five chromosomes. According to the QTL nomenclature, we named the QTL as qPH-A08-(BSA)01, qPH-A08-(BSA)02, up to qPH-A08-(BSA)10 (Fig. 2A and Additional file 9). Although a total of 8 QTL were identified on chromosomes A08, A10, A13, and D03, and Δ (SNP-index) of each QTL just over the threshold line. Thus, we focus on two QTLs that have higher values on chromosome D01, qPH-D01-(BSA)01 and qPH-D01-(BSA)02. The physical position of qPH-D01-(BSA)01 and qPH-D01-(BSA)02 were 6.07–9.66 Mb and 10.69–12.43 Mb, respectively.

### GBS QTL locate in BA F_2_ population

To verify the AA group mapping results, we used another F_2_ population with GBS method to locate the QTL of PH. SLAF sequencing identified a total of 1,543,942 SNPs in two parents. After filtering the SNP site, 7743 marker sites were screened with completeness (75%) and SD (*p* > 0.001). The linkage map was constructed by Joinmap4.0 with 5046 SNP markers in the end. The number of SNP markers and the total genetic distance of each linkage group are shown in Additional file 2 and Additional file 3.The total genetic distance is 5954.87 cM, the longest linkage group length is 713.94 cM, and the maximum gap is 16.94 cM. SNP markers were used from 26 chromosomes and divided into 26 linkage groups, hence, the chromosome number is used instead of the linkage group number. In total, 11 QTL for PH were detected on four chromosomes. Among all QTLs, 6th and 5th QTL were identified on the At and Dt chromosomes respectively (Table 2 and Fig. 2B). On ChrA03, QTL exhibited 15.46% of PVE and 4.8 of LOD between marker mk626_A03 and mk7354_A03. Only *Gh_A03G0832* and *Gh_A03G0833* existed with annotation IPMS2 and RPD1 but showed no differences in expression.

**Table 2:** QTL information for plant height in AA F_2_ group

QTL Name	Chr	Position (genetic)	Position (physical)	LOD	Additive effect	Dominant effect	R2
qPH-A03-(GBS)01	A03	26.5	93,727,847	5.4587	−9.5239	−5.2898	0.0004
qPH-A03-(GBS)02	A03	34.2	93,585,329	5.861	−9.8883	−4.5461	0.001
qPH-A03-(GBS)03	A03	50.1	27,371,589	4.8108	8.5959	−5.3265	0.1546
qPH-A06-(GBS)01	A6	61	93,928,717	3.1788	−4.0743	−3.2641	0.0137
qPH-A06-(GBS)02	A6	338.6	70,513,686	2.5121	−3.585	−2.5447	0.0131
qPH-A06-(GBS)03	A6	597.7	112,971	2.9496	−4.0087	−3.0578	0.0133
qPH-D01-(GBS)01	D01	256.6	25,086,049	3.1118	7.8675	−2.9575	0.0845
qPH-D01-(GBS)02	D01	263.8	22,413,590	4.4199	10.505	−2.2176	0.0976
qPH-D01-(GBS)03	D01	310.1	17,756,478	8.563	−14.693	−3.784	0.0341
qPH-D01-(GBS)04	D01	318.6	17,407,200	8.879	−15.2109	−3.6284	0.0338
qPH-D02-(GBS)01	D02	223.3	44,349,602	2.7979	−4.0764	−2.8479	0.0131
***qPH-D01-(GBS)01***	***D01***	***373.5***	***5,887,414***	***4.3594***	***3.0558***	***−7.2987***	***0***
***qPH-D01-(GBS)02***	***D01***	***385.6***	***6,056,815***	***21.2673***	***12.8405***	***−7.5648***	***0.3529***
***qPH-D01-(GBS)03***	***D01***	***391.3***	***6,023,764***	***23.1031***	***13.061***	***−7.7263***	***0.3611***
***qPH-D01-(GBS)04***	***D01***	***402.7***	***6,698,093***	***5.967***	***11.7556***	***−8.0992***	***0.15***
***qPH-D01-(GBS)05***	***D01***	***433.9***	***9,645,343***	***5.587***	***−6.5948***	***−7.2828***	***0.014***

The blue background reflect the old linkage map QTLs results. The italic and bold font highlight the new D01 linkage map QTLs results

### Comparison analysis of BSA and GBS result

QTL located in chromosome D01 were focused because the first group AA (BSA method) QTL were exactly located on it with a higher e-value. Four QTL qPH-D01-(GBS)01, qPH-D01-(GBS)02, qPH-D01-(GBS)03 and qPH-D01-(GBS)04 were found on D01 with explaining the phenotypic variation of 8.45, 9.76, 3.41 and 3.38% respectively, which can be divided into two QTL clusters. The additive effect of the first QTL cluster is positive, revealing the predominance of male parent Ari1327 on the short stature of the plant.

**Fig. 3:**
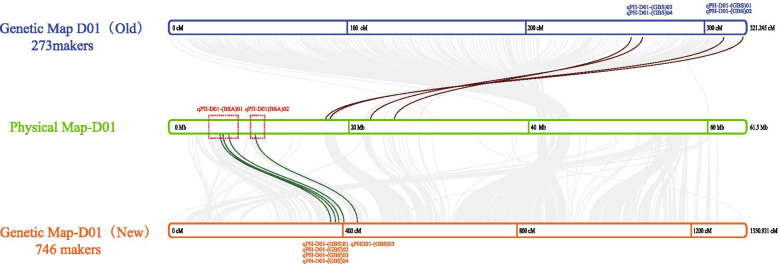
BSA and GBS QTL locate compare analysis. The three from top to bottom are represented as D01 linkage map (Old, 273 markers), D01 physical map (61.5 Mb), and D01 linkage map (New, 746 markers). The line is the collinear relationship between the genetic map and the physical map. The blue QTLs in the top are the mapping cloning sites of the old linkage map, the red QTLs in the physical map represents the BSA-QTL location, and the orange QTLs in the bottom are the mapping cloning sites of the new linkage map

GBS scanned the physical positions of QTL to visualize the overlap area between the two groups. However, D01 chromosome contains 273 SNP markers with a total genetic distance of 321.25 cM and an average genetic distance of 1.18 cM. Moreover, uneven distribution of D01 markers was observed mainly in the front end (Fig. 3). SNPs deficient area was exactly the candidate area identified by group AA (BSA method). However, the physical position in the BA QTL location was still closest to the AA QTL location despite the lack of good markers (Fig. 3 middle, green). So, we re-examined the GBS-D01 markers. It was found that when we performed the chi-square test on the separation ratio of 1:2:1 (*p* < 0.1), after correction (*P* < 0.0001), only 273 markers were obtained, which was the deep orange block on line “marker choose” (Additional file 4). Whether we checked the chi-square value on Additional file 12 or the genotype distribution on Additional file 4 of the AA population, we can find that many markers have been deleted due to statistical differences. So we decided to increase the density of the linkage map by using SDM (segregation distortion markers) to cover the complete chromosome, especially in the front end (Fig. 3 below, orange).

The new linkage map of a single D01 chromosome consists of 746 markers with a total genetic distance of 1330.931 cM. The new linkage map can cover the entire D01 chromosome front and back end as well. Five QTL were found during re-locating with a higher threshold LOD > 3.5. Among them, qPH-D01-(GBS)01, qPH-D01-(GBS)02, qPH-D01-(GBS)03 and qPH-D01-(GBS)04 were responsible for contributing dwarf allele from the male parent Ari1327. These four QTLs range from 0.81 Mb along from 5.89 Mb to 6.7 Mb, thus considered a QTL cluster. The last three QTLs explained the phenotypic variation at 35.29, 36.11 and 15% respectively. qPH-D01-(GBS)05 was located at 9.65 Mb region, which is the second QTL or QTL cluster.

After increasing the linkage map density by using the SDM, the QTL positioning results of BSA and GBS had overlapped. The max candidate area was 5.80 to 9.66 MB on D01from 200 genes, including *Gh_D01G0486* to *Gh_D01G0685.*

### QTL validation by InDel marker

Three InDel markers were used to verify the QTLs, namely p8, p27 and p33 on D01:5818220, 5,930,069 and 6,004,746 bp (Additional file 11). In all 1241 individuals, the ratio of F_1_-like genotype was all more than 50%. The three group’s genotype distribution was beyond the segregation ratio of 1:2:1 (*p* < 0.01). For primer P8, the number of H genotypes was 920, and only 36 individuals were homozygous from the dwarf parent, showing a more severe SD.

**Fig. 4:**
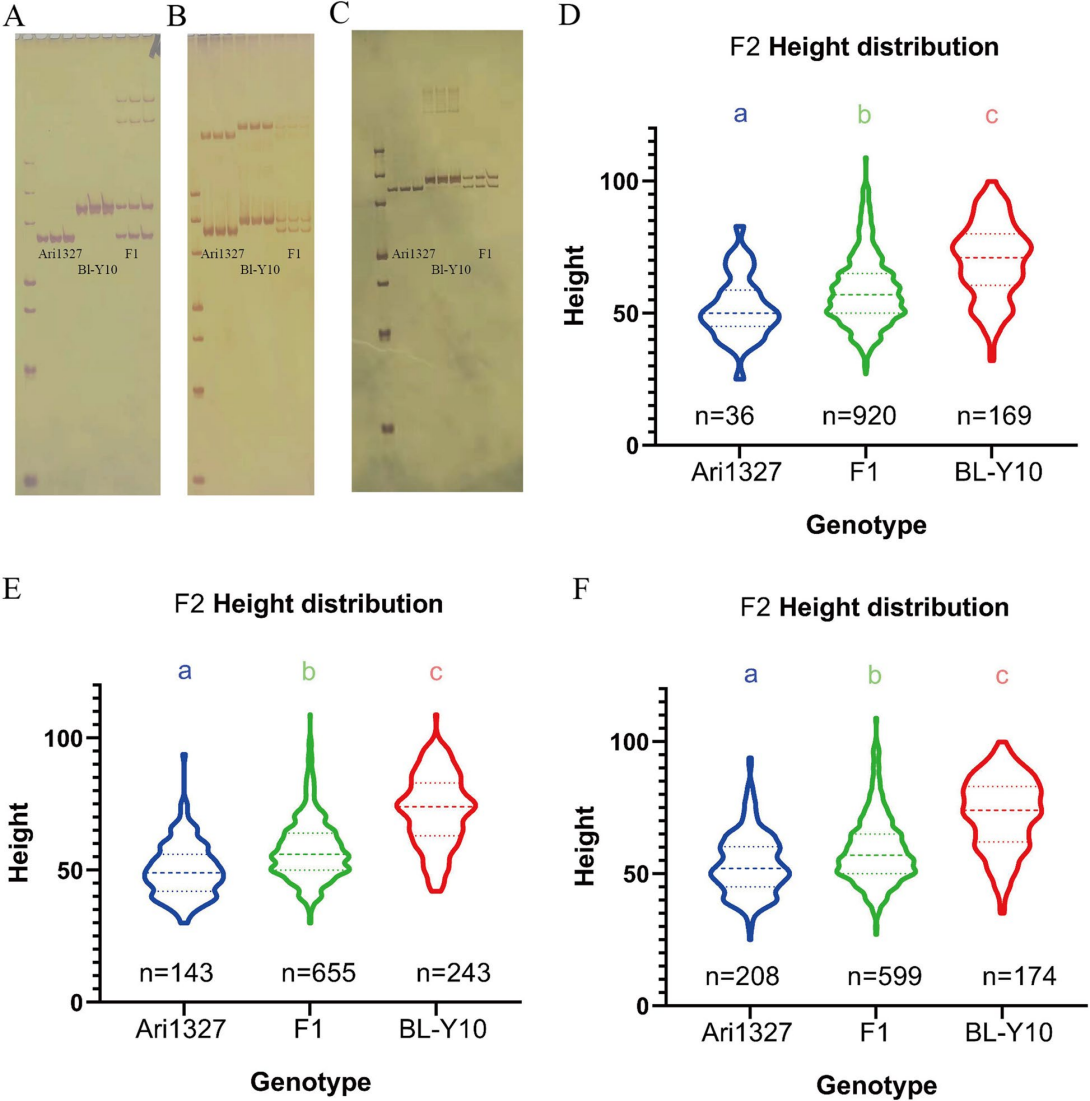
The InDel marker validation. (A), (B), (C) represents the electrophoresis of marker P8, P27 and P33 respectively. The left line is 500 bp marker. From top to bottom, it is 50, 100, 150, 200, 300, 400 and 500 bp. The next three lines are the parent Ari1327, the next three are parent BL-Y10, and the last three are F1 generation. (D), (E), (F) are 1241population genotyping results. The y-axis is the length of plants, and from left to right the x-axis are three genotypes Ari1327, F1 and BL-Y10. The character after “n=” are the number of members in each genotypes. The color characters “a” “b” “c” mean that they are significantly different from each other group (*p* < 0.01)

The estimates of Ari1327 genotype on populations were 52.08 ± 12.03, 50.08 ± 10.23 and53.58 ± 11.42, significantly less than those had BL-Y10 genotype. They had a higher value of 70.07 ± 14.37, 72.67 ± 13.60 and 72.6 ± 13.62 respectively (*p* < 0.01) as shown in Fig. 4. The main QTL that causes Ari1327 to become shorter existed in the candidate area.

### RNA-seq analysis between mutant Ari1327 and wild type Ari971

Although the two QTL mapping methods can successfully co-locate the dwarf QTL on a small interval, still, it contains many candidate genes. To screen of desired gene, gene expression analyses of wild-type and mutant were performed. RNA-seq analysis was carried out in various time intervals for PH of wild-type and mutant plants. Results revealed that both genotypes differed from each at two true leaf stages. The results were more significant at the four true leaves stage, while obvious and highly significant differences in plant height were observed at the six-leaf stage (Fig. S4). Hence, 222, 491 and 1909 DEGs (Differential Expressed Genes) were observed at 2nd, 4th, and 6th leaf stages. 29 DEG existed in the three times but no one existed in the D01 candidate gene area. DEGs at the 2nd leaf stage were not enriched in any pathway, and DEGs at the 4th leaf stage were enriched in four GO pathways and one KEGG pathway. The 6th leaf stage had many DEGs, so all of them can be enriched into 19 GO and 19 KEGG pathways. Four pathways related to the cell cycle and signal pathways like M00693, ko04110, ko04712 and ko04010 were found (Additional file 12). Since, the DEGs shared in the first three time intervals were not in the candidate area, we re-analyzed the expression of all genes (Additional file 12). Genes were selected with FC > |1.5|, FPKM > 0.5, and *P* < 0.01 to construct a heat map. In order to better display the results, the heat map was calculated through log values.

**Fig. 5:**
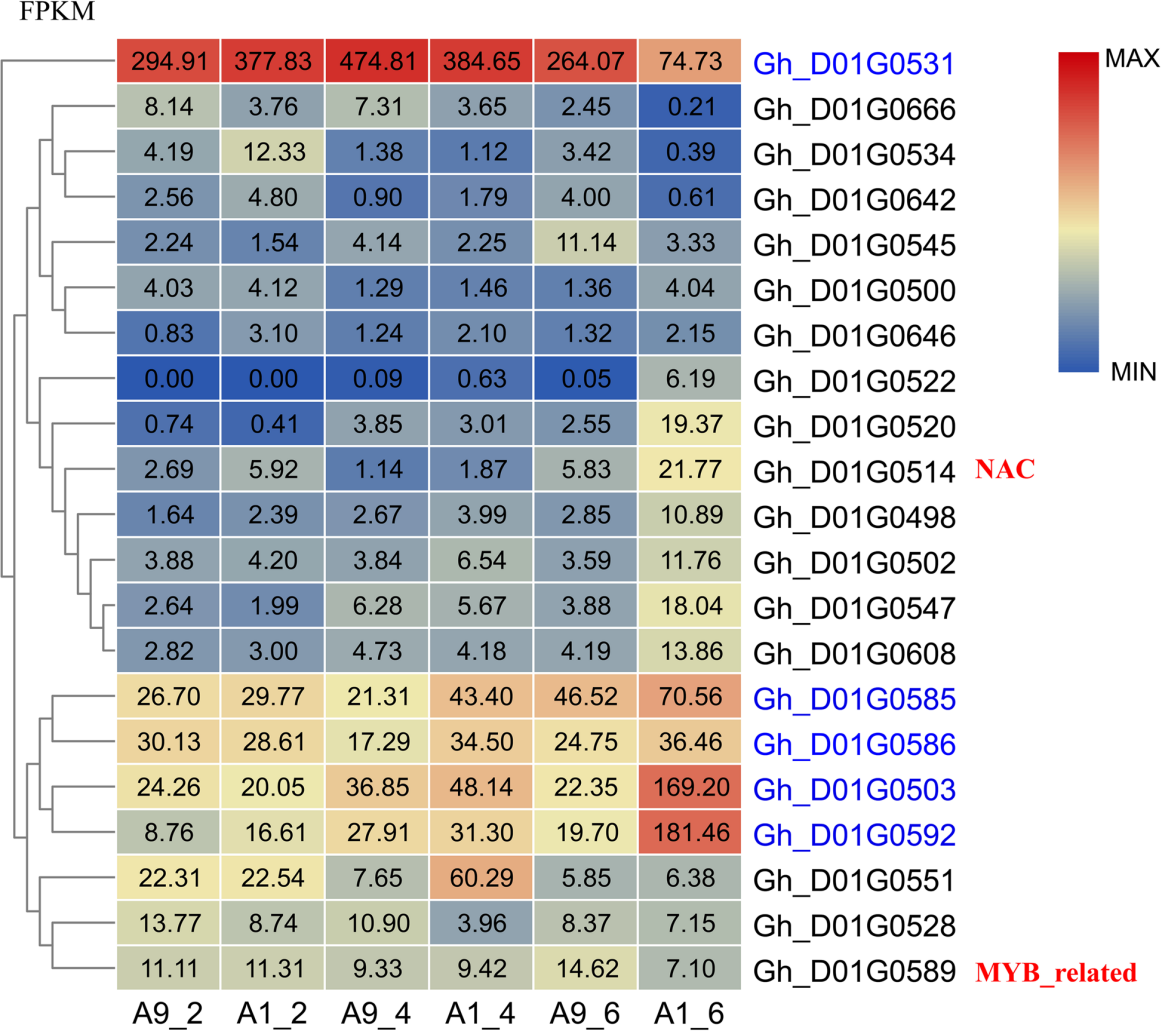
RNA-seq analysis between mutant and wild. FPKM is used for expression, and the number in the bottom A9_2, A1_2, A9_4, A1_4, A9_6 and A1_6 are mean Ari971 for two leaves stage, Ari1327 for two leaves stage, and so on. The red words NAC and MYB_related mean two transcription factors. The blue gene name is most likely the candidate gene

Among the 200 identified genes in the candidate area, 21 genes showed significant differences at least in one time interval. The expression of *Gh_D01G0503*, *Gh_D01G0585*, *Gh_D01G0586* and *Gh_D01G0592* in the mutant was higher than that of wild type during 4th and 6th leaf stages. *Gh_D01G0531* expressed lower at 6th leaf stage in mutant than wild type. Except for these five genes with huge differences, the other 16 genes can be grouped into three clusters according to their expression pattern. The first type contained genes i.e., *Gh_D01G0666*, *Gh_D01G0534*, *Gh_D01G0642*, *Gh_D01G0545*, *Gh_D01G0500* and *Gh_D01G0646* exhibited lower expression values The second class included *Gh_D01G0522*, *Gh_D01G0520*, *Gh_D01G0514*, *Gh_D01G0498*, *Gh_D01G0502*, *Gh_D01G0547* and *Gh_D01G0608*, all of which are expressed higher than wild type at six leaves stage. The other three genes i.e., *Gh_D01G0528*, *Gh_D01G0589* and *Gh_D01G0551* were grouped into third class. Only *Gh_D01G0551* has a notably higher expression at 4th leaf stage in the mutant (Fig. 5).

qRT-PCR was used to verify the expression of the five candidates. These genes all showed significant expression differences in the six leaves stage, *p* < 0.01. The expression trends of *Gh_D01G0503*, *Gh_D01G0531*, *Gh_D01G0585* and *Gh_D01G0592* were consistent with RNA-seq data. *Gh_D01G0503* and *Gh_D01G0592* were highly expressed in the mutant with three to four-fold differences than wild. Furthermore, *Gh_D01G0531* showed almost threefold expression of the wild type. The expression differences of *Gh_D01G0585* and *Gh_D01G0586* were showed 1.5 folds and the expression trend of *Gh_D01G0586* was opposite to RNA-seq.

## Discussion

Semi-dwarf is an essential trait for cotton mechanical picking and yield management [32, 33]. But the discovery and utilization of natural semi-dwarf mutants are still insufficient. Previous studies from our lab have found a semi-dwarf mutant Ari1327 different only in plant height from wild-type Ari971. Preliminary physiological studies exhibited that the length of the mutant’s second and fourth internodes was shorter than wild. However, the number of internodes remain same [34]. In the current study, re-evaluation of the performance of wild-type and mutant were performed based on agronomic traits. Significant differences were observed in FL, the number of sympodial branches, PH, whereas mutant showed short fiber length compared to wild. As we all know, the number of sympodial branches, number of bolls, lint weight (30 bolls) and seed cotton weight (30 bolls) are directly proportional to plant height. Although the yield per plant may decrease with short nature cotton plants, the dwarf population can increase the planting density and the cotton yield per unit area [1].

Moreover, GBS population results in short heighted Ari1327 reinforce no correlation between PH with FL, FU, FS, FE, and MC. Very few dwarf cotton mutants are reported but correlated with fiber or other agronomic traits [11]. Ari1327 is a possible dwarfing germplasm because of the lack of a relationship between dwarfing and fibre quality.

Forward genetics are commonly used to identify the underlying molecular mechanism of a mutant, and QTL-mapping was used to separate populations [35]. However, the grouping of phenotypic data in various environments is a pre-requisite to constructing QTL map. Using RIL (Recombinant Inbred Line), NIL (Near Isogenic Line) and back-cross techniques are great QTL positioning methods, but often take many years to produce multiple generations [31, 36]. Next-generation sequencing (NGS) technology combined with BSA has been proven to be a cost-effective and straightforward rapid QTL detection method. Zhang et al. [37] only used F_1_, F_2_ and BC_1_F_1_ populations to successfully identify maize QTL *qPH7* for plant height. With the advancement in sequencing technology and high-density markers like SNP and tiny-InDel were replacing traditional markers such as RFLP, STS, and SSR in Map-based cloning [38, 39]. In this case, we used F_2_ populations separately to co-locate semi-dwarf genes. Within one year, two populations were produced from the same dwarf parent Ari1327 and different tall parent. High-density genetic linkage map based on SNP marker in another F_2_ population BL-Y10 × Ari1327 was constructed by using enzyme digestion SLAF-seq (Pacific-Locus Amplified Fragment Sequencing). 11 loci are located on four chromosomes, QTL physical position was compared in order to cross-validate BSA-QTL results. However, there is no significant GBS-QTL signal in the BSA-QTL area. The segregation filter process lost a large number of markers at the front end and back end of the chromosome which caused the deviation of QTL position. We re-picked all these SDM and constructed the single D01 chromosome linkage map with higher density. The results of new D01 linkage map provide the better insight and good overlap.

SD is a phenomenon of significant deviations between the observed segregation rate and the expected Mendelian ratio [40, 41], due to gametic selection and interspecific or intraspecific hybridization [42, 43]. SD was related to the proportion of heterozygous alleles in the population or the frequency of heterozygous chromosomes, which affects sex differentiation and gamete selection. It is considered to be an important driving force for biological evolution [44, 45]. SDM was usually deleted in map construction because it will affect the genetic distance and QTL for accuracy [46, 47]. Deleting it may cause potential QTL to be missed, or a change in QTL location [48, 49]. Hackett et al. believed that when the marker density is less than 10 cM, the segregation marker had no significant effect on linkage groups [50]. Zuo et al. used the orthogonal and reciprocal RIL populations of soybean to study the effects of SDM on linkage groups and QTL localization in multiple environments. It is believed that SDM can be added to improve the density and quality of linkage groups [51]. After reconsidering the SDM, repacked all the SDM and had a good QTL overlap with another population. Our results follow the above-mentioned key point that the removal of SDM will reduce the density of linkage groups, even change the position of QTL.

RNA-seq was used to narrow down the selection criteria from 200 candidate genes to 21 genes. Finally, focused on six genes i.e.*, Gh_D01G0503*, *Gh_D01G0514*, *Gh_D01G0585*, *Gh_D01G0586*, *Gh_D01G0503* and *Gh_D01G0592* due to their different expression patterns in three time periods, and the FPKM values are greater than five. Except for *Gh_D01G0586*, the expression trends of the other four genes are consistent with the RNA-seq in the six leaves stage. Among them, *Gh_D01G0585 and Gh_D01G0586* were annotated as homologous genes of the Arabidopsis ubiquitin10. Ubiquitin was involved in regulating many physiological processes of plants including growth and development [52]. However the ubiquitination of proteins mainly depended on E3 ubiquitin ligase to regulate specific signaling pathways [53]. *Gh_D01G0585* and *Gh_D01G0586* genes encode polyubiquitin, and their expression changes should influence the many phenotypes not only PH. The annotation of *Gh_D01G0503* was stem-specific protein. Our laboratory’s previous research on Ari1327 physiology found that internodal shortening was the cause of its dwarfness [34], so stem apex was selected as RNA-seq sampling material. The selection of this gene may be related to our sampling choice, but the possibility of being the target gene was not excluded. *Gh_D01G0531* was annotated as *CAB13*, Chlorophyll a/b-binding protein, or the *LHCB3* protein in the PSII system [54]. In the barley, correlation was elucidated with gene diversity of LHCP protein and the agronomic traits [55]. But in general, there were few related studies about the effect of LHCP protein on dwarfing plant.

*Gh_D01G0514* was one of NAC gene family, which the biggest TF families regulate both development and stress responses [56]. On of AtNACs *JUB1* participates in the negative regulation of GA/BR, to short hypocotyls, dwarfism, late flowering and male sterility [57]. Knockdown of *OsY37* expression caused dwarfism and high accumulation of chlorophyll during the vegetative phase [58]. Wu et al. found *ATAF1*-overexpression lines displayed many altered phenotypes, including dwarfism and short primary roots [59]. In our case, the expression between Ari1327 and Ari971 showed a difference only at the six leaves stage, but it still can be the reason of dwarfing.

Thiamin, also known as vitamin B1, is an essential vitamin for metabolic processes such as the citric acid cycle, glycolysis and pentose phosphate pathway. Thiamine consists of pyrimidine ring and thiazole ring. *THI1* was the first enzymes of thiazole ring synthesize [60]. Thiamine and its active form TPP is an essential cofactor for enzymes involved in a number of critical metabolic processes, such as the production of acetyl-CoA, the tricarboxylic acid cycle (TCA), the pentose phosphate pathway (PPP) and C3 cycle etc. [61]. In *Arabidopsis thaliana*, overexpression of *THI1* appears to increase plant drought resistance and regulate ion channels [62]. In Maize, *THI2* and thiamine played a vital role in proliferation of stem cells [63]. At the same time, thiamine is also reported in DNA repair, metabolism, photosynthesis, and respiration [64, 65]. *Gh_D01G0592* is a kind of THI1, in Ari1327, and it exhibited more expression level than wild type at six leaves stage. The accumulation of excessive thiamine could cause the disturbance of the above-mentioned metabolic cycle, resultsing in chlorosis, growth retardation [66, 67]. Thus, it was prioritized as a dwarfing gene in Ari1327.

## Conclusion

Overwhelmingly, in present study two F2 populations were used to locate the major QTL of the semi-dwarf mutant Ari1327 individually, and successfully co-localizing it to a smaller range on the D01 chromosome. InDel marker phenotyping 1241 population can also confirm the existence of the QTLs. Through RNA-seq and gene annotation, candidate genes were reduced to three from 21 selected genes, i.e., Gh_D01G0503, Gh_D01G0531 and Gh_D01G0592. Among them, Gh_D01G0592 was the key gene, regulating THI1 in the process of thiamine synthesis, and we believed that it is most likely to be the dwarf gene. Therefore, it is suggested that this information must be substantiated by another genetic experimentanother genetic experiment be substantiate this information to verify the gene action. This work will pave the way for understanding the dwarfing mechanism of Ari1327 and finally using its excellent dwarfing genes.

## Methods

### Plant material, mapping population construction and phenotype analysis

The semi-dwarf mutant Ari1327 was produced by ^60^Co γ-ray irradiation treatment of Ari971 [68]. The two tall parental lines Ari3697 and BL-Y10 were crossed as female parents with mutant Ari1327 to generate two F_2_ mapping populations, AA (Ari3697 × Ari1327) and BA (BL-Y10 × Ari1327) in 2018. Ari3697 was the tall height mutant from Ari971. In comparison, BL-Y10 was selected just only by plant height character and not developed from Ari971. Two tall parents have close and distant genetic relationships, respectively. Ari3697 and BL-Y10 were selected as tall parents crossing with Ari1327 in order to observe in generations easily. The AA group of 215 individuals were planted in the experimental area, CRICAAS (Cotton Research Institute, Chinese Academy of Agricultural Sciences), Anyang (Henan Province, China 36.06°N, 114.49°E). While the BA group of 303 individuals were planted in Sanya (Hainan Province, China, 18.41°N, 109.20°E). In addition, we used the same F1 generation of BA group to generate a huge F_2_ validation population of 1241 individual plants in CRICAAS, 2020. All recommended production practices and plant protection measures were adopted during the experiment. Row to row 70 cm and plant to plant distance of 30 cm was maintained. The first dose of fertilizer (DAP, urea) was applied at the germination stage. The second dose of fertilizer (Urea) was applied at the flowering stage. The last fertilizer (Urea) dose was applied at the boll formation stage. Two weeding practices were performed at the cotton field, the first one was performed one month after sowing and the second weeding practice was done after one month of first weeding. The tinning was done with first weeding practices. When the vertical growth ceased, the plant’s final height was measured with a measuring rod from the first cotyledonary node to the apical bud. The sympodial branches or fruit branches are the direct fruit-bearing branches. At maturity, the sympodial branches from each genotype were counted from all selected plants. Also, measure the length of the fruit branches. The number of effective mature bolls from all the picks was counted, and the cumulative record was maintained for each plant separately. Lint percentage or ginning outturn is the weight of lint obtained from a given weight of seed cotton and expressed in percentage. Samples of seed cotton obtained from individual plants were weighed and ginned separately with an electrical ginning machine in the cotton laboratory. Lint was weighed, and values were used for the determination of GOT percentage.

$$\mathrm{GOT}\%=\mathrm{Weight}\ \mathrm{of}\ \mathrm{lint}\ \mathrm{in}\ \mathrm{a}\ \mathrm{sample}/\mathrm{Weight}\ \mathrm{of}\ \mathrm{seed}\ \mathrm{cotton}\ \mathrm{in}\ \mathrm{a}\ \mathrm{sample}\times \kern0.37em 100$$

Seed cotton weight is the 30 bolls weight. 30 cotton bolls were picked and seeds were taken at random for each sample from each genotype and weighed. Similarly, lint of picked 30 bolls was also weighed separately for lint weight. The fiber characters were measured using high volume instrument 900 (HVI-900, Uster technologies Ltd., Switzerland). It is a computerized instrument that provides a comprehensive profile of raw fiber. Agronomic and fiber traits between Ari1327 and wild species were recorded by a core collection of upland cotton project. This two-year experiment was planned in six environments with three blocks and 16 agronomic traits (Table 1) [69]. Ari1327 and wild species were also grown in the greenhouse for RNA-seq. Genomic DNA was extracted using CTAB (Cetyltrimethylammonium bromide) method [70]. The stem tips of wild-type and mutants were used to extract RNA with RNAprep pure Plant Kit DP441 (Tiangen Biotech, Beijing, China). Statistical analysis for plant height was conducted using SPSS (Version 23.0, SPSS, Chicago). In this project, all the seeds Ari1327, Ari971, BL-Y10 and Ari3697 were provided by Germplasm Repository of Institute of Cotton Research, Chinese Academy of Agricultural Sciences (CRI of CAAS, Anyang, Henan province, China) only for scientific research purposes.

### QTL mapping by BSA

The two DNA pools were developed by mixing an equal amount of DNA from the 20-maximum height (Higher than 87.05 cm) and 20 dwarfs (lower than 60 cm) lines of AA F_2_ populations which were screened by BSA (bulked segregant analysis). Both DNA pools were randomly broken into fragments at the size of around 350 bp by the method of ultrasonication and sequenced by Illumina HiSeq 2500 (Biomarker Technology, Qingdao, China). The experimental process was carried out according to the standard protocol provided by Illumina [71]. The raw reads were trimmed and filtered, then aligned to the TM-1 (*G. hirsutum*) [72] reference genome using the BWA software [73]. Aligned files were converted to BAM format and detected SNPs using SAMtools [74] and GATK software [75]. ANNOVAR software [76] was used to annotate SNPs. After identifying polymorphic SNP markers between parents, the two lines have already been re-sequenced in the core collection of the upland cotton project [69]. SNP-index and Δ (SNP-index) were calculated by sliding window method with default settings of 1 Mb and step size of 10 kb. Only the area where Δ (SNP-index) above the threshold level of 0.01 significance were considered candidate loci [77, 78].

### QTL mapping by GBS

The BA group was screened using GBS (Genotyping-by-sequencing) to construct an ultra-high-density genetic map. MseI and NlaIII were used to double digest the genomic DNA of F_2_ populations and their parents. Furthermore, the libraries were constructed and sequenced on Illumina HiSeq PE150 platform according to the manufacturer’s standard protocols. The method mentioned in section 2.2 was used to reads filter, alignment and SNPs detection. The SNPs sites where no homozygous genotype is detected in any of the two parents, firstly filtered out, and then further screened those sites on the basis of mark integrity (75%) and SD (segregation distortion) (*p* < 0.001). Joinmap4.0 [79] was used to construct a linkage map (Maximum likelihood) and WinQTLCart2.5 [80] was employed to map QTL (CIM mapping method, window size set at 10 cM, LOD > 2.5, 1000 permutation, 1 cM walking speed) [81, 82]. The genotype was coded as two from the female parent. So that when the additive effect is positive, female (BL-Y10) in the parental allele provides the effect of increasing plant height, and male (Ari1327) provides the effect of reducing plant height [83]. In addition, using the marker for un-screened by SD to construct the single linkage map of D01 chromosome.

### QTL validation

InDel markers in the candidate area were used for QTL validation. The principle of InDel selection was first homozygous and different between two parents, second the sequence difference was between 5 to 100 bp and finally the sequencing depth must be greater than 10. Once the sites were determined, the 250 bp of its upstream and downstream flanking sequence was used to design primer on NCBI Primer-BLAST tools (https://www.ncbi.nlm.nih.gov/tools/primer-blast). PCR reactions contained 2 μl of genomic DNA template (50 ng/μl), 5 μl of 3G Taq Master Mix for PAGE (Vazyme, Nanjing, China), 0.25 μl primer (100 μM/μl) × 2 and 2.5 μl water. PCR steps are followed as: 95 °C for 5 min; 35 cycles of 95 °C for 15 s, 50–65 °C for 15 s, 72 °C for 30 s; and a final extension at 72 °C for 5 min. The PCR amplification products were separated on 8% polyacrylamide gel by electrophoresis of 200 V for 2.0 h. The gel was stained in 0.1% AgNO3 solution (Sinopharm Chemical Reagent, Shanghai, China), and revealed the DNA bands in 1.5% sodium hydroxide and 0.4% formaldehyde solutions (Sinopharm Chemical Reagent, Shanghai, China) [84]. According to the genotype consistent with the parents or F1, the plant height data of 1241 populations were divided into three groups. ANOVA-test and Chi-square were performed at (*p* < 0.01).

### RNA-seq and qRT-PCR validation between wild and mutant

The critical period in which the mutant showed a high degree of difference was measured and set a sampling point before and after it. RNA extraction was carried out using an RNAprep Pure Plant Kit (TIANGEN Biotech, BeiJing, China). Then samples were sent to the company (Biomarker Technology, Qingdao, China) for RNA-sequencing and data analysis was performed in Biocloud online platform (Biomarker Technology, QingDao, China). HISAT2 [85] and StringTie [86] aligned and assembled the fileted reads to TM-1 reference genome. The FPKM (fragments per kilobase of transcript per million fragments mapped) was calculated to find the area of candidate DEGs (differential expressed genes). The condition for differential genes was *p*-value < 0.01, FC (fold change) ≥ |1.5|. Enrichment analysis was conducted on CottonFGD website (https://cottonfgd.org/), the significance level was 0.0001, and the minimum gene number for each analyzed term was five. The extracted RNA was also used for qRT-PCR, 1 μg of total RNA was used to be transcribed into cDNA by using One-Step RT-PCR Kit (Novoprotein Scientific, BeiJing, China). The qPCR was performed by using ChamQ Universal SYBR qPCR Master Mix (Vazyme Biotech co., ltd, NanJing, China). LightCycler 480by Roche Diagnostics GmbH, Mannheim, Germany was used to perform PCR amplification. Detailed Primer information used in the current study is given in Table S3.

## Supplementary Information

**Additional file 1.**

**Additional file 2.**

**Additional file 3.**

**Additional file 4.**

**Additional file 5.**

**Additional file 6.**

**Additional file 7.**

**Additional file 8.**

**Additional file 9.**

**Additional file 10.**

**Additional file 11.**

**Additional file 12.**

**Additional file 13.**

## Data Availability

All raw data was submitted to NCBI (https://www.ncbi.nlm.nih.gov/sra).The accession number of GBS data is PRJNA734430. The original data for parent BL-Y10 of BSA is part of project PRJNA634606, and the internal number is GH0543. The raw data of parent Ari1327 and extreme pools of BSA is PRJNA734972. The accession number of RNA-seq between Ari1327 and Ari971 is PRJNA733290.

## Abbreviations

QTL: quantitative trait locus
BSA: bulk segregation analysis
GBS: genotyping by sequencing
DEG: differential expressed genes
FL: average length of upper fibers
FU: fiber uniformity
MC: micronaire
FS: fiber strength
FE: fiber elongation
PH: plant height
SDM: segregation distortion markers
SD: segregation distortion
NIL: Near Isogenic Line
RIL: Recombinant Inbred Line
