# Supplementary figures and images for: Mapping of dwarfing QTL of Ari1327, a semi-dwarf mutant of upland cotton
Source: BMC Plant Biol. 2022 Jan 3;22:5. doi: 10.1186/s12870-021-03359-x (PMC8722190; doi:10.1186/s12870-021-03359-x)

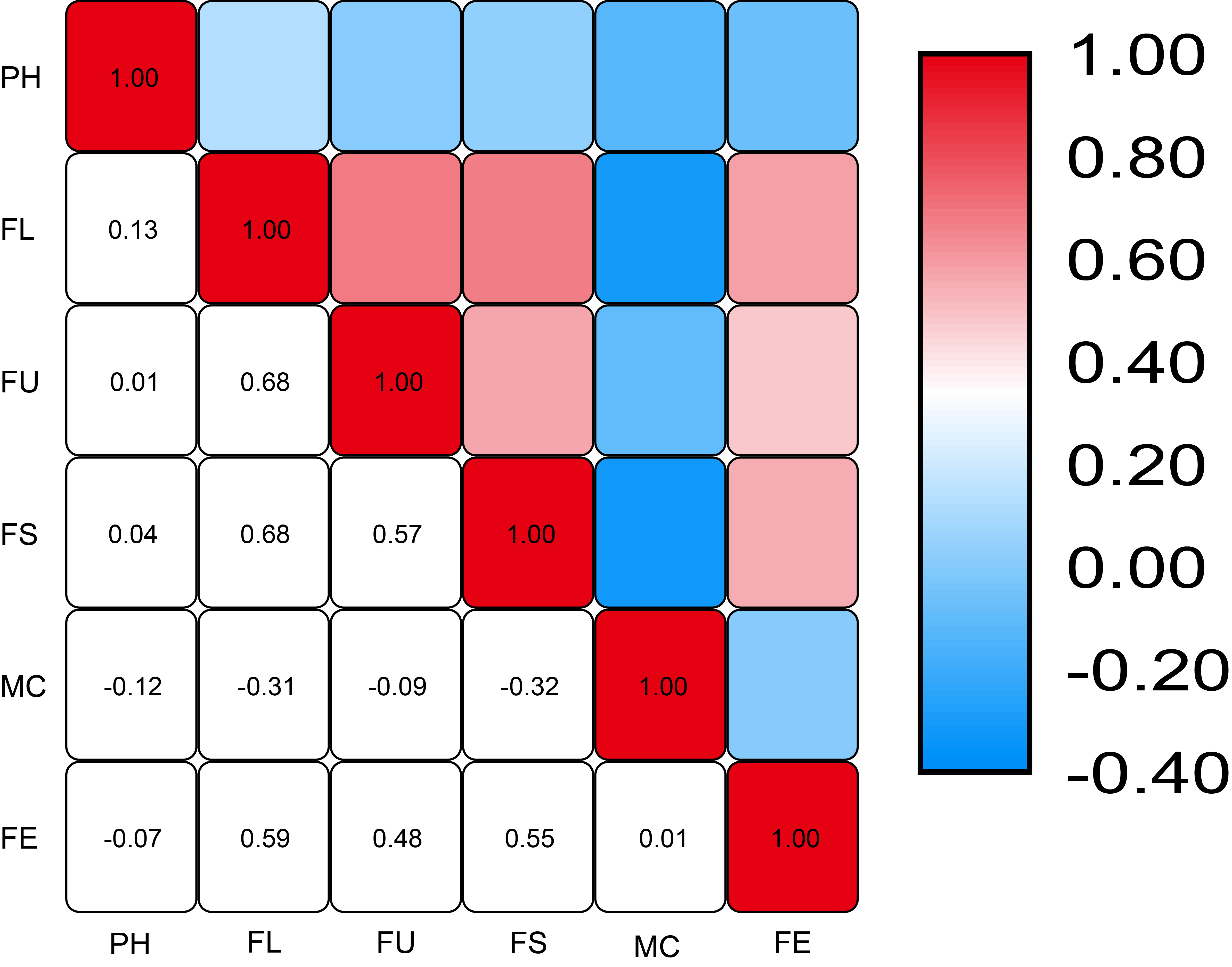

Supplement: Supplementary file 1 — Additional file 1. [file 12870_2021_3359_MOESM1_ESM.jpg]

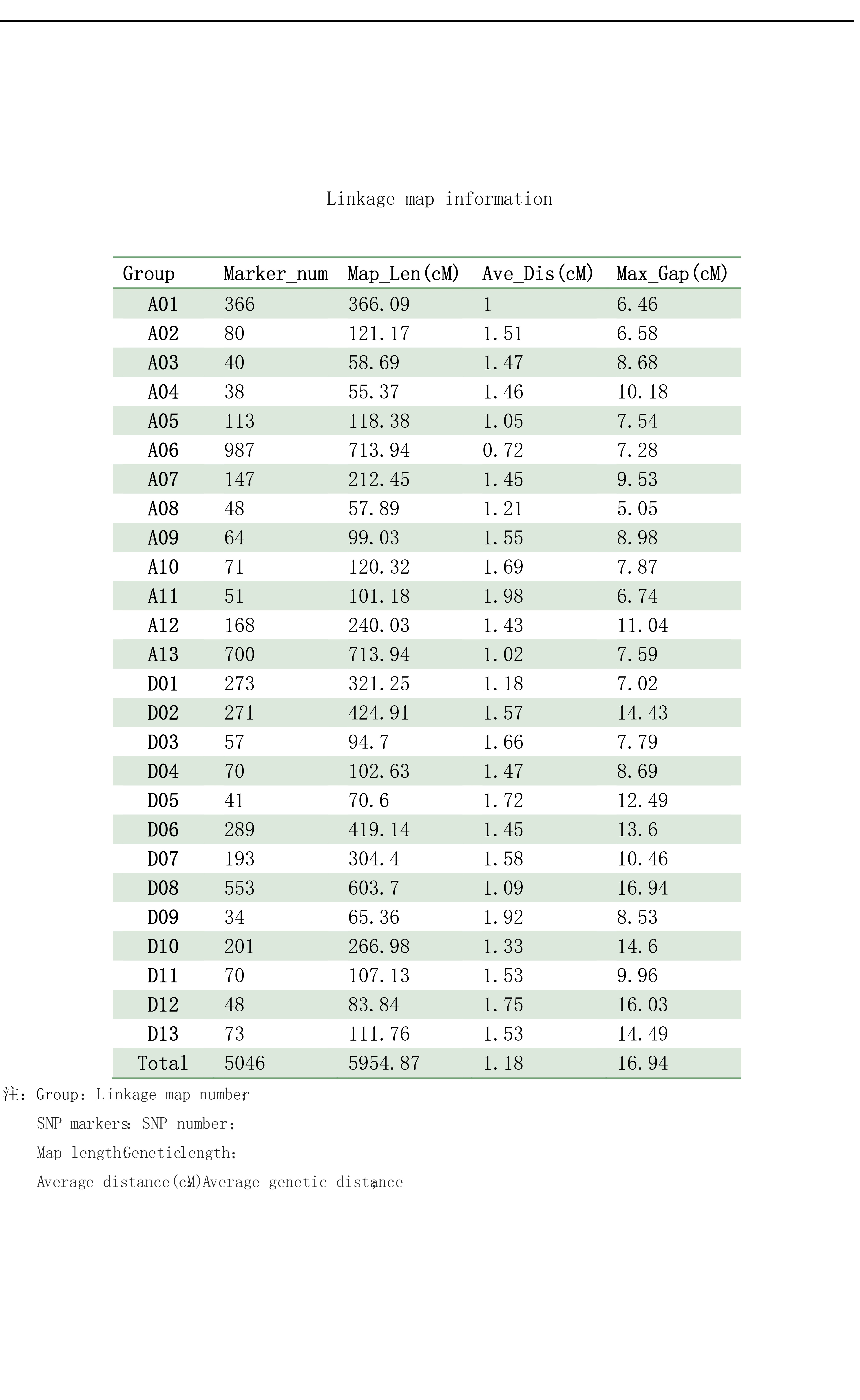

Supplement: Supplementary file 3 — Additional file 3. [file 12870_2021_3359_MOESM3_ESM.jpg]

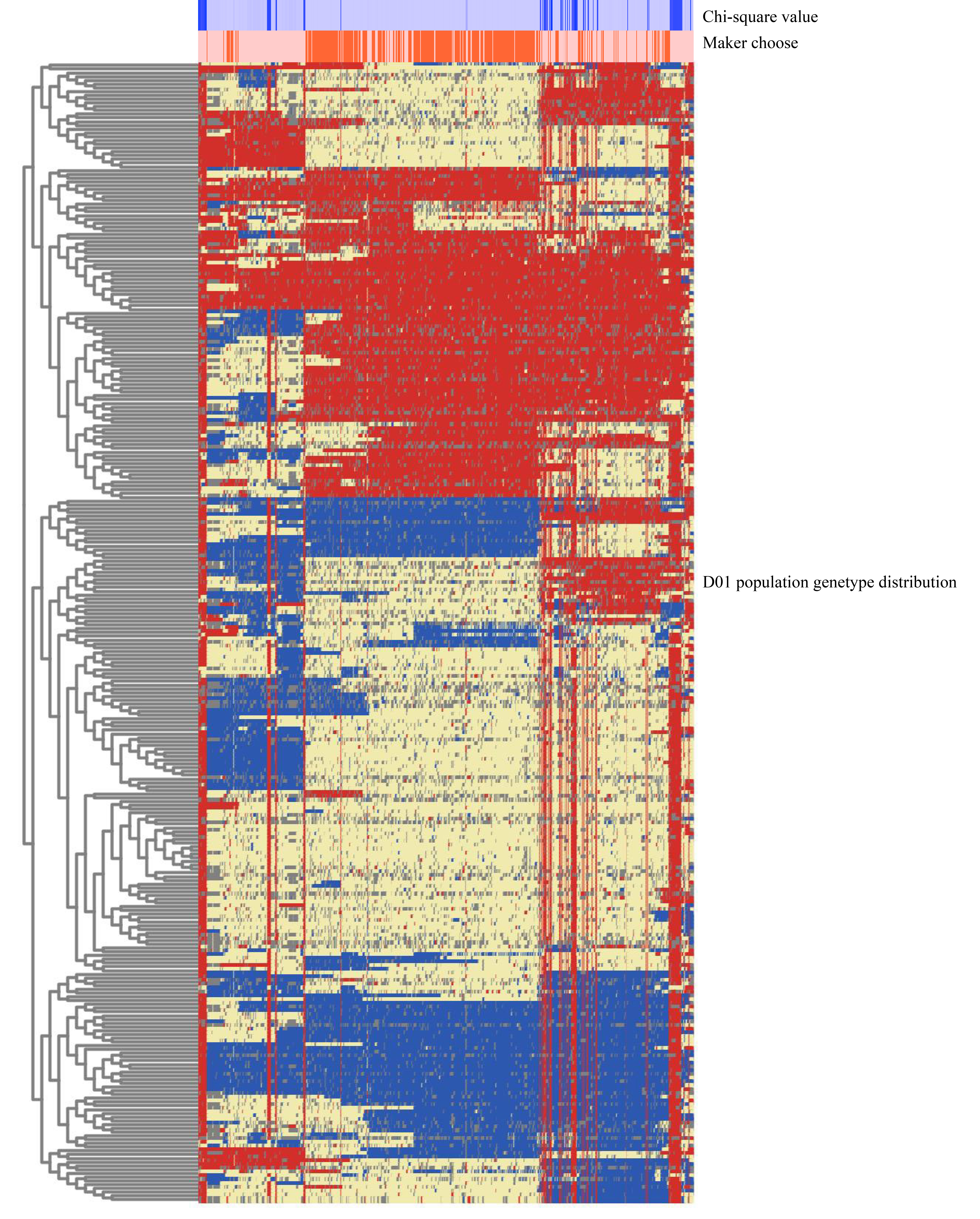

Supplement: Supplementary file 4 — Additional file 4. [file 12870_2021_3359_MOESM4_ESM.jpg]

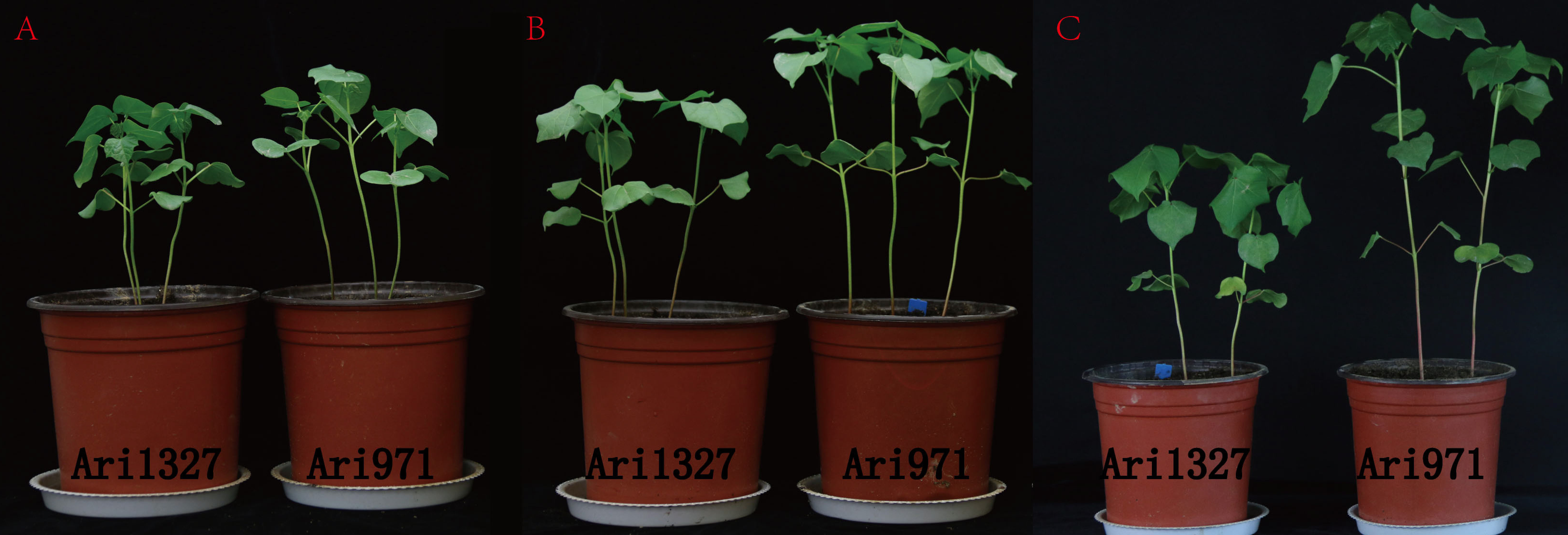

Supplement: Supplementary file 5 — Additional file 5. [file 12870_2021_3359_MOESM5_ESM.jpg]

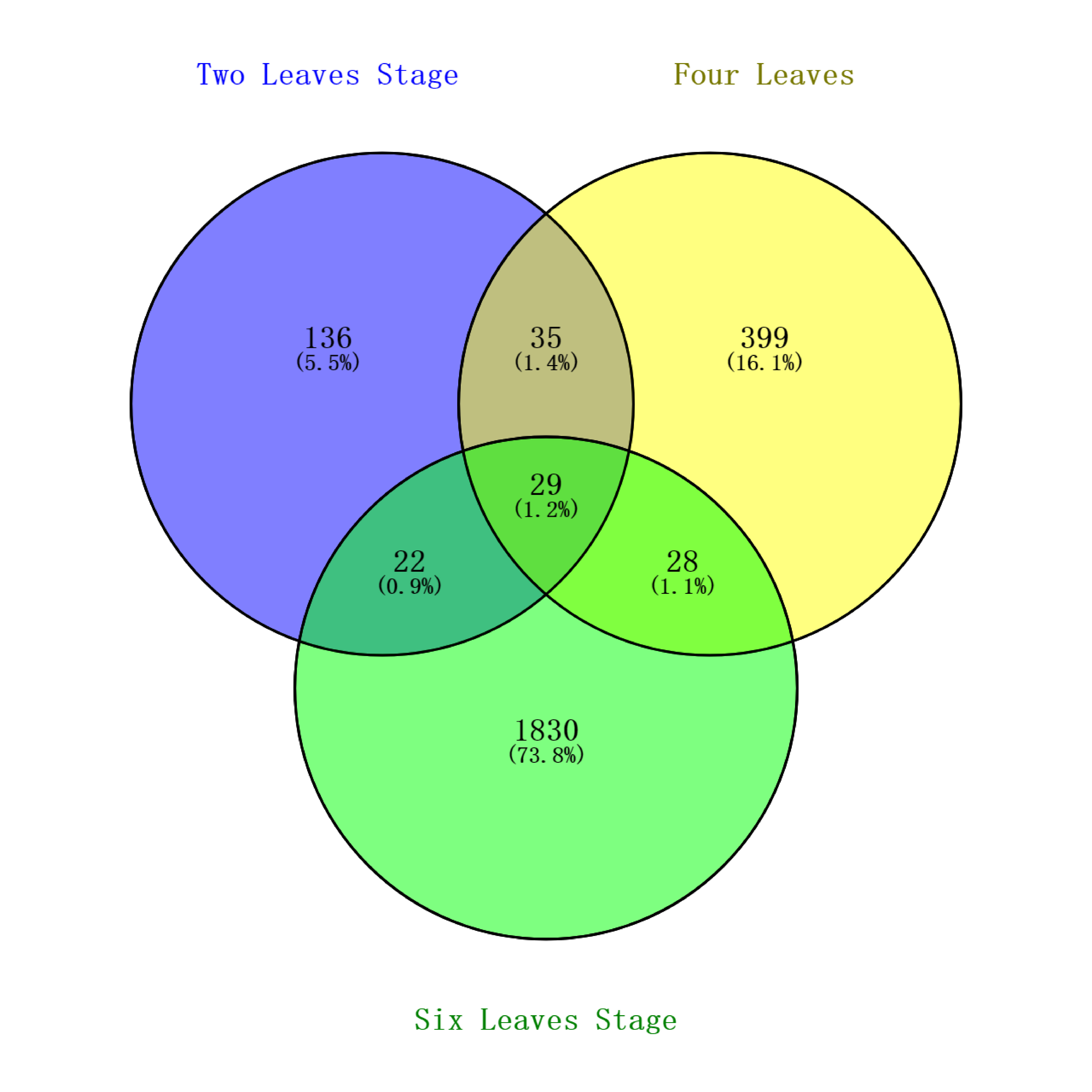

Supplement: Supplementary file 6 — Additional file 6. [file 12870_2021_3359_MOESM6_ESM.jpg]

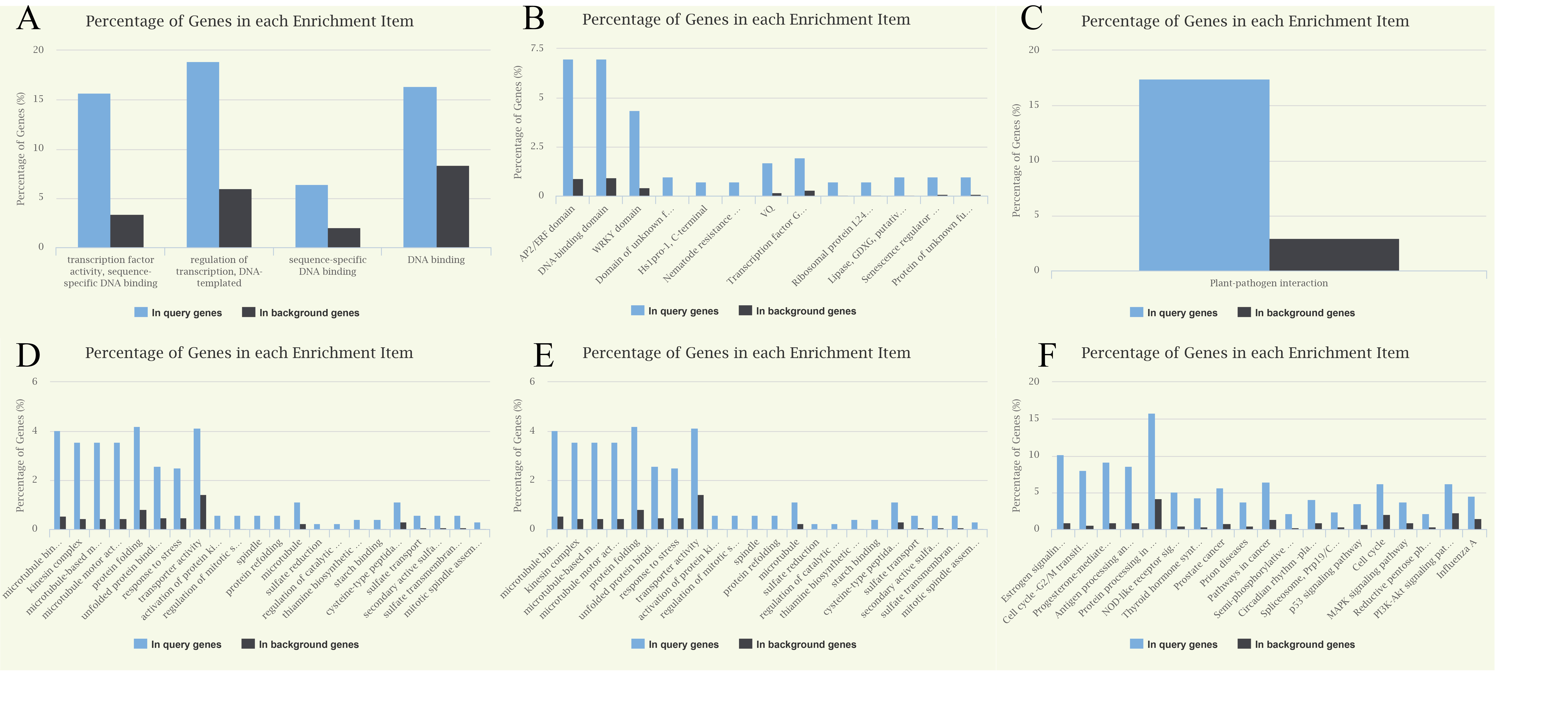

Supplement: Supplementary file 7 — Additional file 7. [file 12870_2021_3359_MOESM7_ESM.jpg]

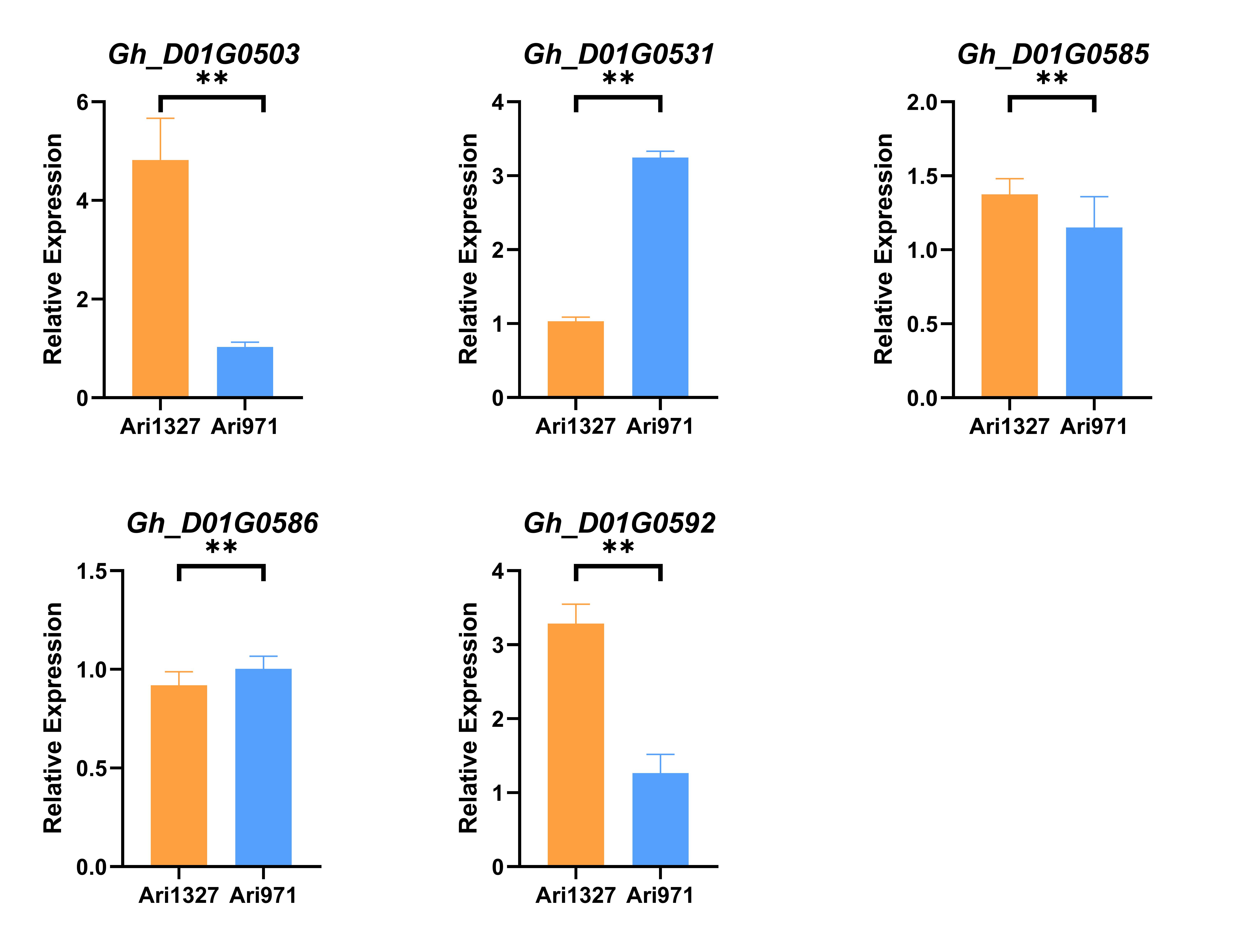

Supplement: Supplementary file 8 — Additional file 8. [file 12870_2021_3359_MOESM8_ESM.jpg]
