# Supplementary material for: Mapping of dwarfing QTL of Ari1327, a semi-dwarf mutant of upland cotton
Source: BMC Plant Biol. 2022 Jan 3;22:5. doi: 10.1186/s12870-021-03359-x (PMC8722190; doi:10.1186/s12870-021-03359-x)
Supplement: Supplementary file 13 — Additional file 13. [file 12870_2021_3359_MOESM13_ESM.docx]

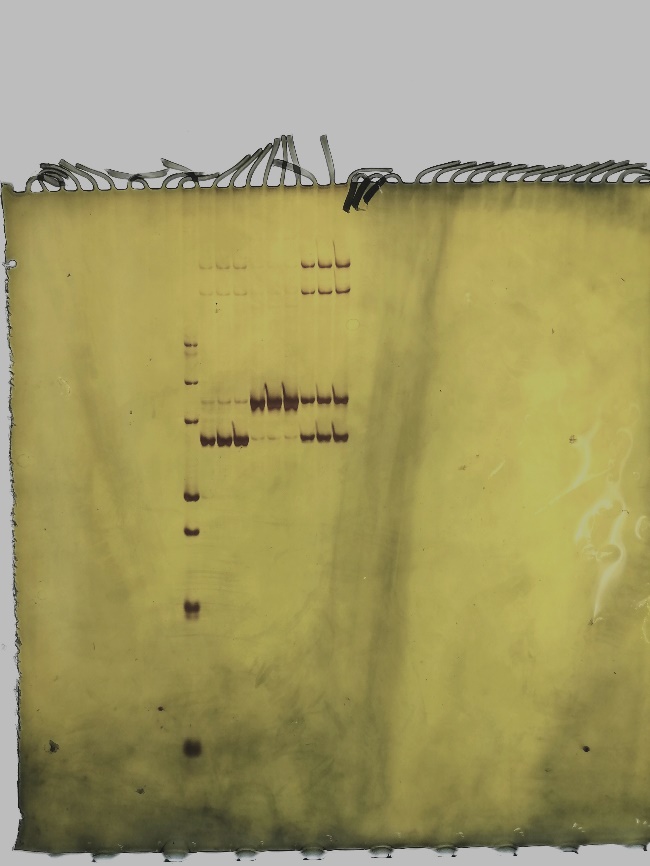


Maker Ari1327 BL-Y10 F1

Primer P8 validation. The left line is 500bp maker. From top to bottom, it is 50, 100, 150, 200, 300, 400 and 500bp. The next three lines are the parent Ari1327, the next three are parent BL-Y10, and the last three are F1 generation.


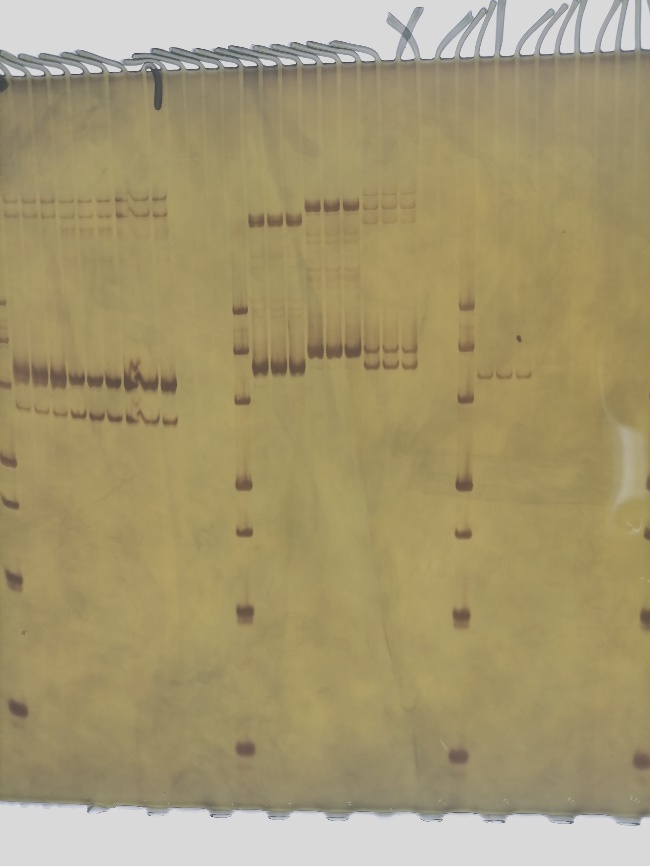


Maker Ari1327 BL-Y10 F1

Primer P27 validation. The left line is 500bp maker. From top to bottom, it is 50, 100, 150, 200, 300, 400 and 500bp. The next three lines are the parent Ari1327, the next three are parent BL-Y10, and the last three are F1 generation.


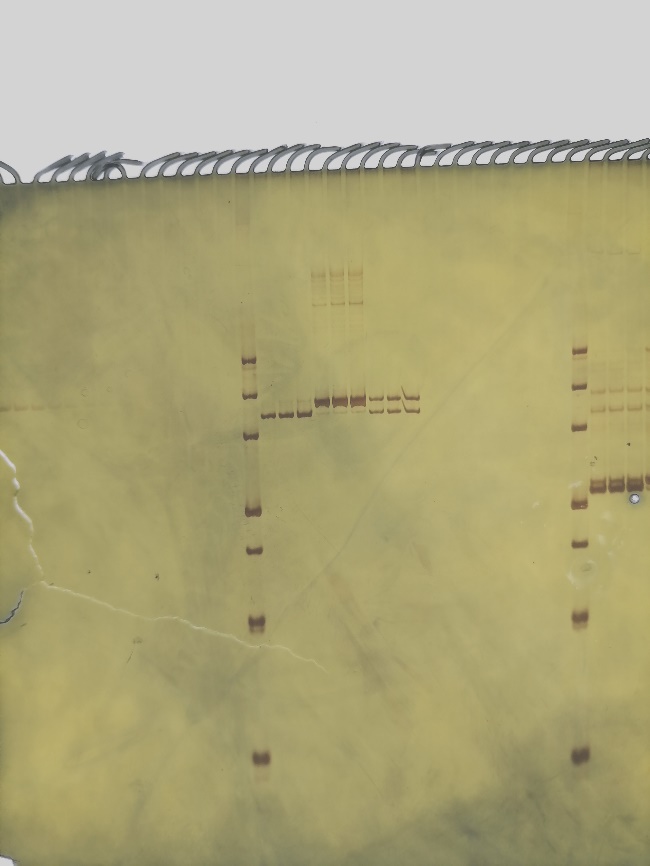
Primer P33 validation. The left line is 500bp maker. From top to bottom, it is 50, 100, 150, 200, 300, 400 and 500bp. The next three lines are the parent Ari1327, the next three are parent BL-Y10, and the last three are F1 generation.

Maker Ari1327 BL-Y10 F1
